# Accelerometer-Derived Patterns of Physical Activity and Incident Frailty

**DOI:** 10.1093/geroni/igab046.1299

**Published:** 2021-12-17

**Authors:** Amal Wanigatunga, Yurun Cai, Jacek Urbanek, David Roth, Jeremy Walston, Karen Bandeen-Roche, Lawrence Appel, Jennifer Schrack

**Affiliations:** 1 Johns Hopkins Bloomberg School of Public Health, Baltimore, Maryland, United States; 2 Johns Hopkins School of Medicine, Baltimore, Maryland, United States; 3 Johns Hopkins University, Baltimore, Maryland, United States; 4 Johns Hopkins University School of Medicine, Baltimore, Maryland, United States

## Abstract

Low physical activity (PA) is a common phenotype of frailty, but whether disengagement of daily lifestyle PA signals impending frailty remains unexplored. Using STURDY (Study to Understand Fall Reduction and Vitamin D in You) data from 499 robust/prefrail adults (mean age=76 + 5 years; 42% women), we examined whether accelerometer patterns (activity counts/day, active minutes/day, and activity fragmentation) were prospectively associated with incident frailty over 2 years of follow-up; 48 (10%) participants developed frailty. In Discrete-Cox hazard models adjusted for demographics, medical conditions, and device wear days, every 30 min/day higher baseline active time, 100,000 more activity counts/day, and 1% lower activity fragmentation was associated with a 13% (p=0.003), 10% (p=0.001), and 8% (p<0.001) lower risk of frailty, respectively. Our results show that both reduced amounts and fragmented patterns of daily PA captured from accelerometry are associated with phenotypic frailty and might signal frailty onset.

